# Authors’ response: importance of a careful investigation to avoid attributing Legionnaires’ disease cases to an incorrect source of infection

**DOI:** 10.2807/1560-7917.ES.2020.25.34.2001570

**Published:** 2020-08-27

**Authors:** Claudia Palazzolo, Gaetano Maffongelli, Alessandra D’Abramo, Luciana Lepore, Andrea Mariano, Antonella Vulcano, Tommaso Ascoli Bartoli, Nazario Bevilacqua, Maria Letizia Giancola, Enrico Di Rosa, Emanuele Nicastri

**Affiliations:** 1 National Institute for Infectious Diseases ‘Lazzaro Spallanzani’ IRCCS, Rome, Italy; 2 Local Health Office, ASL Roma 1, Rome, Italy

**Keywords:** waterborne infections, bacterial infections, Legionnaires' disease, Legionella, infection control

**To the editor:** We thank MC Rota, M Scatturo and ML Ricci for their letter with consideration and research interest in our article and we fully agree with their comments [1]. 

It is true that we were not able to find the source of infection of the *Legionella* pneumonia case described in our study, but we know that for *Legionella* sporadic or isolated cases the finding of the origin of the causative pathogen is rarely pursued [2].

There is no evidence that the source of infection in our case was at the workplace and indeed some authors stipulate that home water systems are likely to be linked to sporadic Legionnaire's disease (LD) [3]. Our results, do not support this hypothesis. First, if people are locked down staying at home and home water systems are linked to sporadic cases, an increasing number of LD cases should be notified during not after lockdown. This was not the case in the area of Rome concerned by our study. Second, if people are at home, home water systems are frequently used, and low amounts of *Legionella*, if present in freshwater, would not usually be expected to cause disease [4].

Nevertheless, our aim was to alert clinicians and public health officials about LD cases at the time of the end of lockdown in all countries where this is now declared. LD needs to be considered in the differential diagnosis of patients with acute respiratory syndrome and adequate LD prevention and control measures need to be in place for all buildings that were closed during lockdown.
